# All eyes on FOXC2

**DOI:** 10.7554/eLife.90747

**Published:** 2023-08-10

**Authors:** Wei Yan, John R McCarrey

**Affiliations:** 1 Lundquist Institute for Biomedical Innovation at the Harbor-UCLA Medical Center Torrance United States; 2 Department of Medicine, David Geffen School of Medicine at UCLA Los Angeles United States; 3 Department of Neuroscience, Developmental and Regenerative Biology, University of Texas at San Antonio San Antonio United States

**Keywords:** spermatogonial stem cell, FOXC2, spermatogenesis, quiescent, recovery, testis, Human, Mouse

## Abstract

New evidence in mice suggests that cells expressing the transcription factor FOXC2 may form a reservoir of quiescent stem cells that contributes to sperm formation.

**Related research article** Wang Z, Jin C, Li P, Li Y, Tang J, Yu Z, Jiao T, Ou J, Wang H, Zou D, Li M, Mang X, Liu J, Lu Y, Li K, Zhang N, Yu J, Miao S, Wang L, Song W. 2023. Identification of quiescent FOXC2+ spermatogonial stem cells in adult mammals. *eLife* **12**:RP85380. doi: 10.7554/eLife.85380.

Male fertility relies on the continuous production of sperm via a process known as spermatogenesis. This involves spermatogonial stem cells (SSCs) dividing to form undifferentiated spermatogonia (uSPGs), which then progress through the meiotic and haploid phases of spermatogenesis to form mature sperm (de Rooij, 1998). To ensure that the supply of sperm remains constant, SSCs must continuously provide new uSPGs while also self-renewing to maintain their stocks.

While the existence of SSCs in the adult testis is undisputed, their origin, identity and maintenance remain unclear. In fact, scientists still lack genetic markers that clearly allow them to distinguish these cells from the rest of the uSPG pool. So far, the hallmark feature of SSCs is their ability to re-establish full spermatogenesis when transplanted into testes devoid of germ cells (Kubota and Brinster, 2018; Lord and Oatley, 2018).

Previous work has identified three types of uSPGs – single, paired and aligned – which emerge during the first phase of the differentiation process. When a single uSPG divides, it can sometimes produce paired daughter cells that remain connected after mitosis. In turn, these paired uSPGs can expand to form chains of four to 32 aligned uSPGs, with some of these cells progressing through to the later stages of spermatogenesis to form mature sperm (de Rooij, 2017; Kubota and Brinster, 2018; de Rooij, 1998).

Transplantation experiments have revealed that most cells which can perform the hallmark feature of SSCs (that is, re-establishing full spermatogenesis in testes lacking germ cells) are found within the single uSPG population, but may also be present among paired and aligned progenitors (Kubota and Brinster, 2018). Meanwhile, genetic studies combined with lineage-tracing experiments have highlighted several genes predominantly expressed in single uSPGs that act as SSCs; however, these genes cannot represent strict SSC markers as they are also expressed in progenitors engaged in the differentiation process (Kubota and Brinster, 2018; Sharma et al., 2019). Now, in eLife, Wei Song and colleagues at the University of Dundee and the Peking Union Medical College – including Zhipeng Wang as first author – report findings which suggest that a transcription factor known as FOXC2 may represent a more precise marker of functional SSCs (Wang et al., 2023).

The team started by screening the expression profile of individual cells in a population of mouse uSPGs containing both SSCs and progenitors. Among the top ten genes preferentially enriched in these cells, *Foxc2* was the only one to code for a protein exclusively present in the nucleus of uSPGs that also expressed ZBTB16, a protein important for SSCs to self-renew. A closer look showed that *Foxc2* expression was most abundant in single uSPGs compared to paired or aligned uSPGs. Interestingly, FOXC2-producing uSPGs were mostly quiescent, with only 5% featuring markers associated with proliferation. This finding is consistent with the fact that many FOXC2-regulated genes are involved in cell cycle arrest.

To test whether FOXC2-producing uSPGs could underpin spermatogenesis, Wang et al. transplanted a population of uSPGs enriched in these cells into the testes of mice treated with busulfan, a toxic compound that kills endogenous germ cells. After two months, these animals had generated a much larger number of colonies of differentiating cells compared to control mice which had received a non-enriched uSPG population. Based on these results, Wang et al. set out to show that FOXC2-producing single uSPGs are in fact functional SSCs.

The first step was for the team to follow the fate of these cells for six weeks following transplantation. This revealed that this population could give rise to all subtypes of uSPGs, with some of the resulting progenitors differentiating into sperm that could fertilise eggs and generate offspring. However, FOXC2-producing uSPGs were also capable of self-renewal, forming cells which feature genetic markers associated with SSCs. More specifically, the lineage-tracing experiments showed that FOXC2-producing uSPGs could produce paired uSPGs that would then either divide to form two single uSPGs (including some that retained *Foxc2* expression), or form chains of aligned uSPGs containing at most one FOXC2-producing cell (Figure 1A).

**Figure 1.**
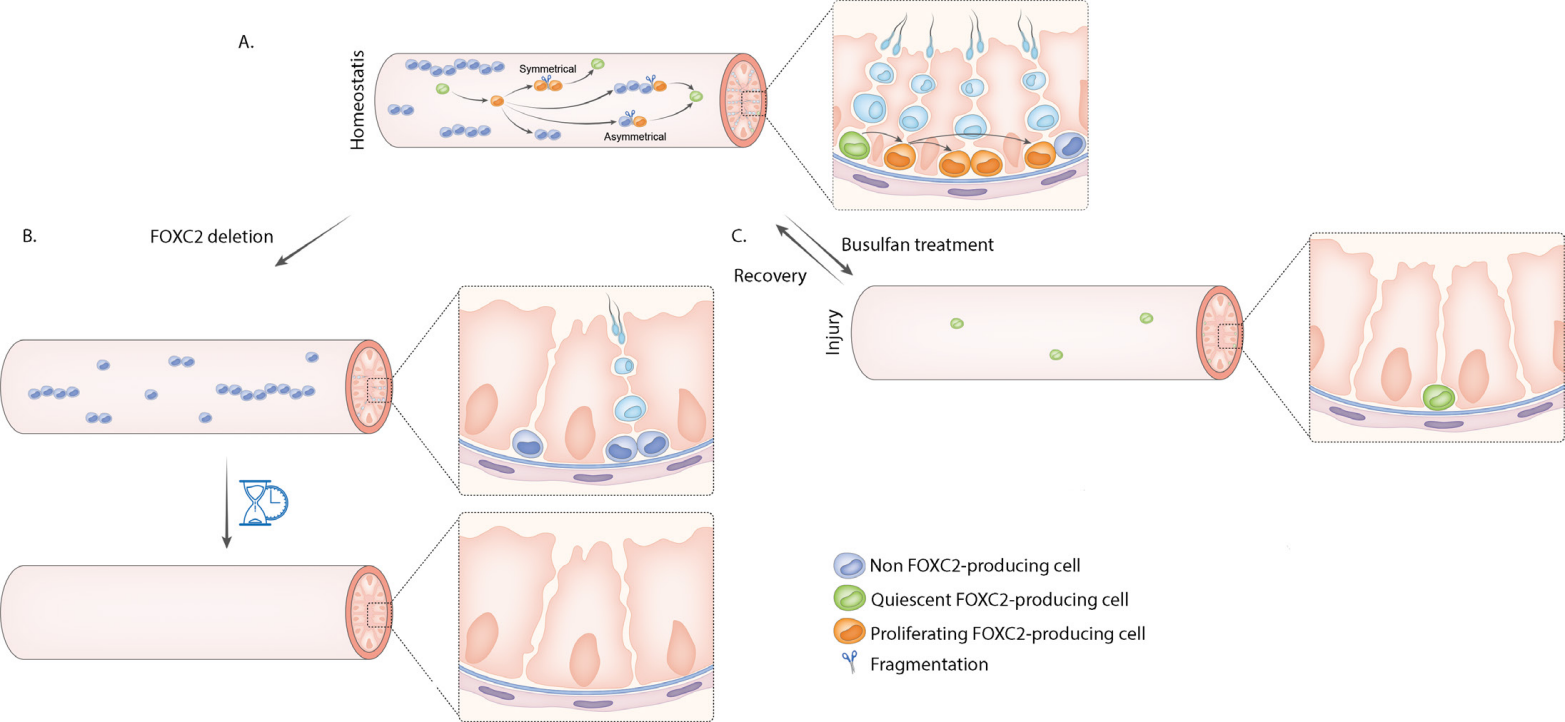
Undifferentiated spermatogonia which express *Foxc2* may represent the entire population of spermatogonial stem cells in the adult testes of mice. (**A**) Sperm is created inside seminiferous tubules (pink tubes) through spermatogenesis, a complex differentiation process that starts with the division of spermatogonial stem cells (SSCs). SSCs are difficult to distinguish from other types of undifferentiated spermatogonia (uSPGs) which also reside in the seminiferous tubules. Wang et al. propose that single uSPGs that express the gene encoding the transcription factor FOXC2 constitutes most, if not all, the SSC pool. About 95% of these cells are quiescent (green cells) and the remaining ~5% are proliferative (orange cells). Proliferating FOXC2-producing single uSPGs may divide asymmetrically to generate paired and aligned uSPGs that remain attached to each other after mitosis. These cells show differential expression of *Foxc2.* Some of the cells that do not produce FOXC2 (light blue cells with dark blue nucleus) will differentiate into progenitors (light blue cells) and ultimately become sperm. Others, which have retained *Foxc2* expression, may split away from their sister cells through a fragmentation process (scissors) and return to a FOXC2-producing single uSPG state to contribute to the SSC pool. Self-renewal of SSCs may also be achieved by symmetrical division of a single FOXC2-producing SSC to form two single, FOXC2-producing daughter SSCs. (**B**) Deleting FOXC2-producing cells causes accelerated exhaustion of SSCs, and, in time (hourglass) leads to male infertility. (**C**) Quiescent FOXC2-producing single uSPGs are resistant to cytotoxins such as busulfan treatment; they can survive these environmental disruptions and replenish the pools of uSPGs, thereby maintaining SSC homeostasis.

Wang et al. then inactivated *Foxc2* in the germ cells of adult testes to better investigate FOXC2 function. This gradually exhausted the number of available uSPGs, leading to smaller testes and eventual infertility (Figure 1B). If *Foxc2* was deleted in male germ cells before mice started to produce sperm, however, an initial wave of spermatogenesis was still able to occur but without subsequent, continuous sperm production. This is consistent with the fact that the first wave of sperm cell formation does not rely on SSCs, while subsequent spermatogenesis does.

Finally, Wang et al. tested whether FOXC2-producing uSPGs contribute to germline regeneration, an important property that allows sperm production to resume after being disrupted. They exposed adult mice to busulfan and found that the remaining population of uSPGs was primarily formed of quiescent FOXC2-producing cells; this aligns with previous findings showing that quiescence helps to protect stem cells from environmental insults (Murley et al., 2022; Tümpel and Rudolph, 2019). After a month, FOXC2-producing cells showed signs of higher levels of proliferation (yet the size of the population remained stable), and after four months spermatogenesis had been fully re-established (Figure 1C).

Together, these results suggest that single uSPGs which express *Foxc2* could indeed constitute the reservoir of SSCs in the mammalian testis. According to these findings, FOXC2 may promote a reversible quiescent state through negative regulation of cell cycle progress. However, a small fraction of this population (~5%) undergoes active proliferation, creating a number of paired and then aligned uSPGs which may include a single cell that continues to express *Foxc2*. Such *Foxc2*-expressing cells may detach themselves from their sister cells in pairs or chains, returning to a single uSPG state and contributing to the renewal of the SSC pool. Meanwhile, other paired and aligned uSPGs that are not expressing *Foxc2* progress through spermatogenesis to form sperm.

Overall, this work provides strong evidence that FOXC2 could mark functional SSCs more precisely while also actively shaping the fate of these cells. This transcription factor is highly conserved and, as Wang et al. show, it is expressed in a similar pattern in human and mouse testes (Wei et al., 2018). FOXC2 may therefore emerge as a useful marker and important regulator for investigating fertility issues in men.
